# Supplementary material for: Venomix: a simple bioinformatic pipeline for identifying and characterizing toxin gene candidates from transcriptomic data
Source: PeerJ. 2018 Jul 31;6:e5361. doi: 10.7717/peerj.5361 (PMC6074769; doi:10.7717/peerj.5361)
Supplement: Supplemental Information 3 [file peerj-06-5361-s003.gz › FinalOutput_E-6/Snaclec_bothroalternin_subunit_alpha@beta_7/finaltree.pdf]

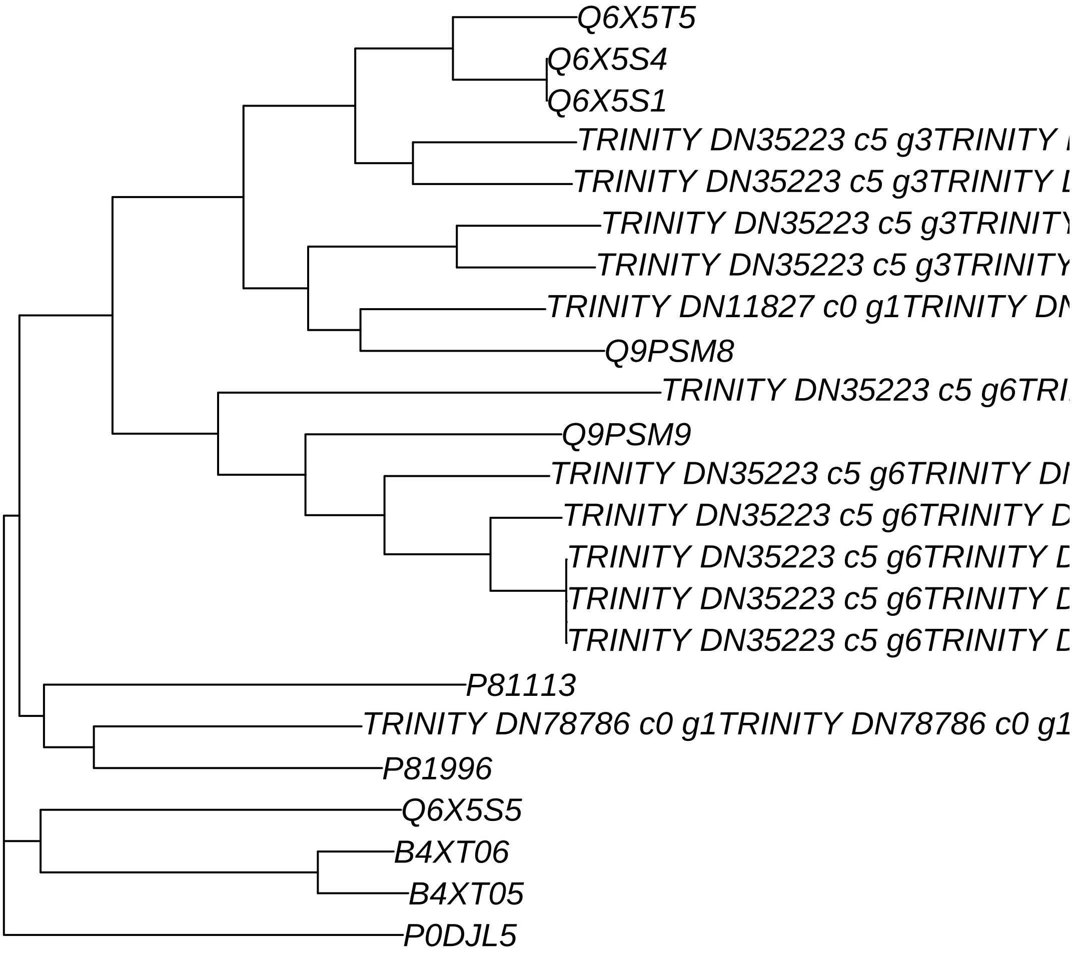

Q6X5T5
Q6X5S4
Q6X5S1
TRINITY DN35223 c5 g3TRINITY
TRINITY DN35223 c5 g3TRINITY
TRINITY DN35223 c5 g3TRINITY
TRINITY DN35223 c5 g3TRINITY
TRINITY DN11827 c0 g1TRINITY
Q9PSM8
TRINITY DN35223 c5 g6TRI
Q9PSM9
TRINITY DN35223 c5 g6TRINITY
TRINITY DN35223 c5 g6TRINITY
TRINITY DN35223 c5 g6TRINITY
TRINITY DN35223 c5 g6TRINITY
TRINITY DN35223 c5 g6TRINITY
P81113
TRINITY DN78786 c0 g1TRINITY DN78786 c0 g1
P81996
Q6X5S5
B4XT06
B4XT05
P0DJL5
